# Posterolateral Versus Posteromedial Approach for Posterior Malleolus Fixation in Trimalleolar Fractures of the Ankle

**DOI:** 10.7759/cureus.69402

**Published:** 2024-09-14

**Authors:** Ashwinkumar Khandge, Rahul Salunkhe, Amit Kale, SomiReddy Medapati, Pankaj Sharma, Ketan Kulkarni, Rishyendra Varma

**Affiliations:** 1 Orthopedics, Dr. D. Y. Patil Medical College, Hospital and Research Centre, Dr. D. Y. Patil Vidyapeeth (Deemed to be University), Pune, IND

**Keywords:** american orthopedic foot and ankle society (aofas) ankle /hindfoot score, ankle fracture fixation, posterior malleolus fracture, posterolateral approach, posteromedial approach, trimalleolar fracture

## Abstract

Background

Ankle fractures, particularly those involving the posterior malleolus, are prevalent injuries that can lead to significant functional impairment if not managed appropriately. The choice of surgical approach for posterior malleolus fixation - posteromedial (PM) or posterolateral (PL) - remains a debate among orthopedic surgeons. The PM approach is a traditional technique involving extensive soft tissue dissection, while the PL approach offers improved visualization and precision with potentially less soft tissue disruption.

Materials and methods

This prospective comparative study was conducted at the Department of Orthopedics, Dr. D. Y. Patil Hospital and Research Centre, Pune, between February 2022 and August 2024.A total of 42 patients with trimalleolar fractures involving the posterior malleolus were randomly assigned to either the PL or PM surgical groups, with 21 patients in each group. Surgical outcomes were assessed using the American Orthopedic Foot and Ankle Society (AOFAS) scores, articular surface step-off, range of motion (ROM), and complications such as osteoarthritis, infection, neurovascular injury, nonunion, and deep vein thrombosis at six months, 12 months, and final follow-up.

Results

Patients in the PL group demonstrated superior clinical outcomes with higher mean AOFAS scores at six-month (87.52 ± 2.92) and 12-month (90.28 ± 1.72) follow-ups compared to the PM group (84.95 ± 3.25 at six months; 88.86 ± 2.41 at 12 months), with statistically significant differences favoring the PL approach. However, as per the final follow-up, the difference in AOFAS scores between the two groups was not statistically significant. The PL group also had more patients with excellent ROM and fewer complications, such as arthritis, than the PM group.

Conclusion

The PL approach for posterior malleolus fixation in trimalleolar fractures offers superior early functional outcomes and fewer complications than the PM approach. However, the long-term outcomes between the two approaches show no significant difference, indicating that both techniques can be effective depending on the specific fracture pattern and patient characteristics.

## Introduction

According to reports, there are 187 cases of ankle fractures for every 100,000 person-years, making it the fourth most prevalent injury that orthopedic surgeons treat [1]. Among ankle fractures, the incidence rate of posterior malleolar fracture is 7-44% and seldom occurs in isolation [1,2]. It accompanies fractures of one or both malleoli and often results from ankle rotation. Rupture of the posterior inferior tibiofibular ligament (PITFL) or the posterior tibial margin avulsion fractures, also known as Volkmann's fracture or posterior malleolus fracture, can occur due to this rotational force [3]. In 1932, Henderson was the first to use the phrase "trimalleolus fracture" [4]. The majority of surgeons believe that to prevent posttraumatic osteoarthritis (OA) of the ankle joint and to create a stable ankle, fragments of posterior malleolus, including more than 25-30% of the distal tibial plafond surface, should be corrected [5,6]. Langenhuijsen *et al*. observed that achieving anatomical alignment of the posterior fragment with a well-reduced joint surface is advisable when more or equal to 10% of the articular surface of the tibia is affected [7]. Recent research indicates a shift from focusing solely on posterior fragment size to prioritizing anatomical joint restoration and addressing intra-articular step-off [8]. Still, there remains debate in orthopedic surgery over the optimal treatment approach for posterior malleolus fractures [8]. Reduction and fixation with lag screws or plating is the effective method to attain favorable functional recovery by achieving anatomical realignment in unstable ankle fractures, thereby preventing OA of the ankle joint [9-11]. A posterior approach, either posterolateral (PL) or posteromedial (PM), is recommended for the posterior malleolus [1].

The PM method has been used with proven results. However, there are problems with significant soft tissue stripping to reach the posterior malleolus fragment through a long medial incision along the tibialis posterior [1]. Recently, reports on the PL method have been made for posterior malleolar fractures; a long J-shaped incision that begins posterior to the fibula border proximally and then bends around the lateral malleolus and ends distally across the dorsolateral foot is used [1]. The PL approach is becoming more favored due to its improved visualization and ability to achieve accurate anatomical reduction, which has shown promising results for treating posterior malleolar fractures [12]. It simplifies treatment by allowing for the simultaneous repair of distal fibula and posterior tibial rim fractures with a single incision [5,13].

In the PM approach, overstretching the soft tissues might cause damage to the tibialis posterior artery or nerve [1]. Furthermore, when partial crossing of the threads of the lag screw through the fracture line, anteroposterior screws might not be able to generate appropriate interfragmentary compression [1]. In contrast, fragments of the posterior malleolus can be directly seen using the PL technique, which also helps with precise fracture reduction and repair using buttress plates or lag screws [1].

Clinical studies have indicated favorable outcomes with the PM approach for specific posterior malleolar fracture patterns [14]. The posteromedial and posterolateral approaches have advantages and disadvantages, yet comparative studies between these techniques still need to be made available. Reliance solely on plain lateral radiographs for fragment size measurement may be unreliable when fragments are rotationally displaced. Therefore, Bois *et al*. proposed a new measurement method utilizing computed tomography (CT) scans to evaluate posterior fragment size accurately [15]. The longstanding "one-third rule" for fixation has largely been discarded due to an enhanced understanding of the three-dimensional nature of these fractures and insights from clinical and biomechanical studies [8]. Consequently, indications for fixation have undergone significant evolution. Restoration of tibiotalar articular congruency by plafond impaction and fragment reduction (or the removal) has been the main focus, along with incisura fibularis reestablishment for syndesmotic stabilization and appropriate reduction of the distal fibula. These guidelines serve as the modern benchmarks for treatment plans [8].

The study compares the clinical outcomes of the PM and the PL approaches: the reduction and fixation of posterior malleolus in a trimalleolar fracture. The objective is to know the functional outcome and complications in both PM and PL approaches for posterior malleolus fixation of the trimalleolar fracture. Therefore, this research aims to treat posterior malleolus fractures by contrasting the established PM technique with the relatively recent PL approach.

This study hypothesizes that the PL approach for posterior malleolus fixation in trimalleolar ankle fractures will result in superior clinical outcomes compared to the PM approach. Specifically, it is anticipated that patients treated with the PL approach will demonstrate higher American Orthopedic Foot and Ankle Society (AOFAS) scores, better range of motion (ROM), and more precise fracture reduction, leading to improved functional recovery.

## Materials and methods

This is a prospective comparative study from February 2022 to August 2024, and we had 42 cases, 21 each in the PM and PL groups. The study was conducted at the Department of Orthopedics, Dr. D. Y. Patil Hospital and Research Centre, Dr. D. Y. Patil Vidyapeeth (Deemed to be University), Pune, after getting approval from the Ethical Committee (IESC/PGS/2022/102). All investigations and procedures were done only if clinically indicated. No specific or additional investigations were done for the study. Most of the investigations/procedures were done free of cost; any investigation/procedure/implant indicated clinically was borne by the patient, as per the hospital’s policy. We do not have any conflict of interest to disclose.

Inclusion criteria included patients aged 18-65 years with confirmed trimalleolar fractures involving the posterior malleolus (Mason classification type 2B) who were operated on within 1-2 weeks of injury after the swelling subsided.

Exclusion criteria included patients with open fractures, tibial pilon fractures (AO/OTA 43-C type), pathological fractures, or those with severe comorbidities that might affect healing, and patients who were not able to bear weight on the affected side (not due to trauma).

**Figure 1:**
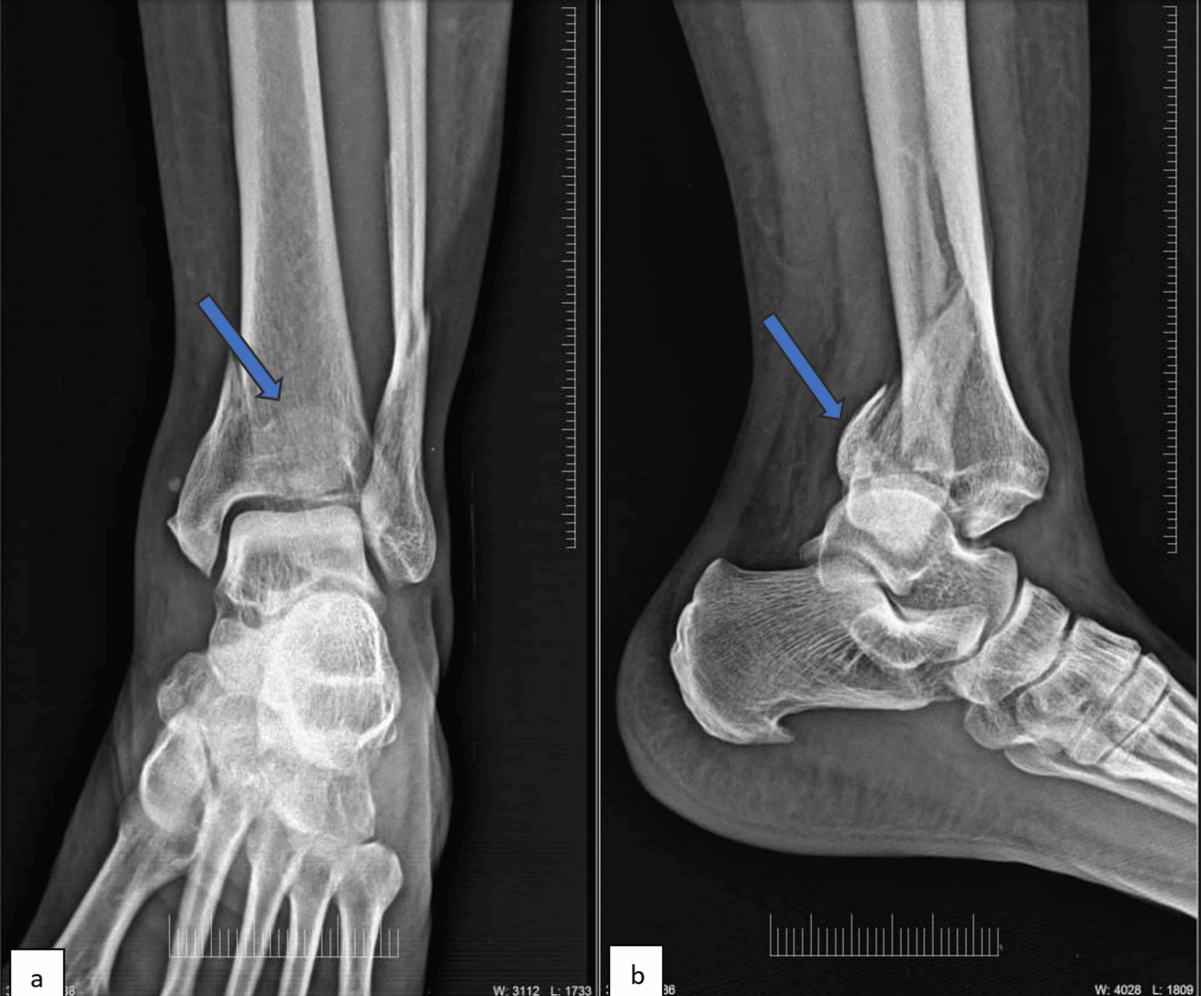
X-ray of an ankle in (a) anteroposterior and (b) lateral view This is a representative X-ray of preoperative trimalleolar fracture in the PM group with posterior malleolus marked. PM: Posteromedial

The fractures were Mason type 2B, which could be approached by either PL or PM. So, patients were randomly assigned to the PL or PM group (Figure 1) using a computer-generated randomization sequence. This ensured an equal distribution of patients across both groups, minimizing selection bias.

**Figure 2:**
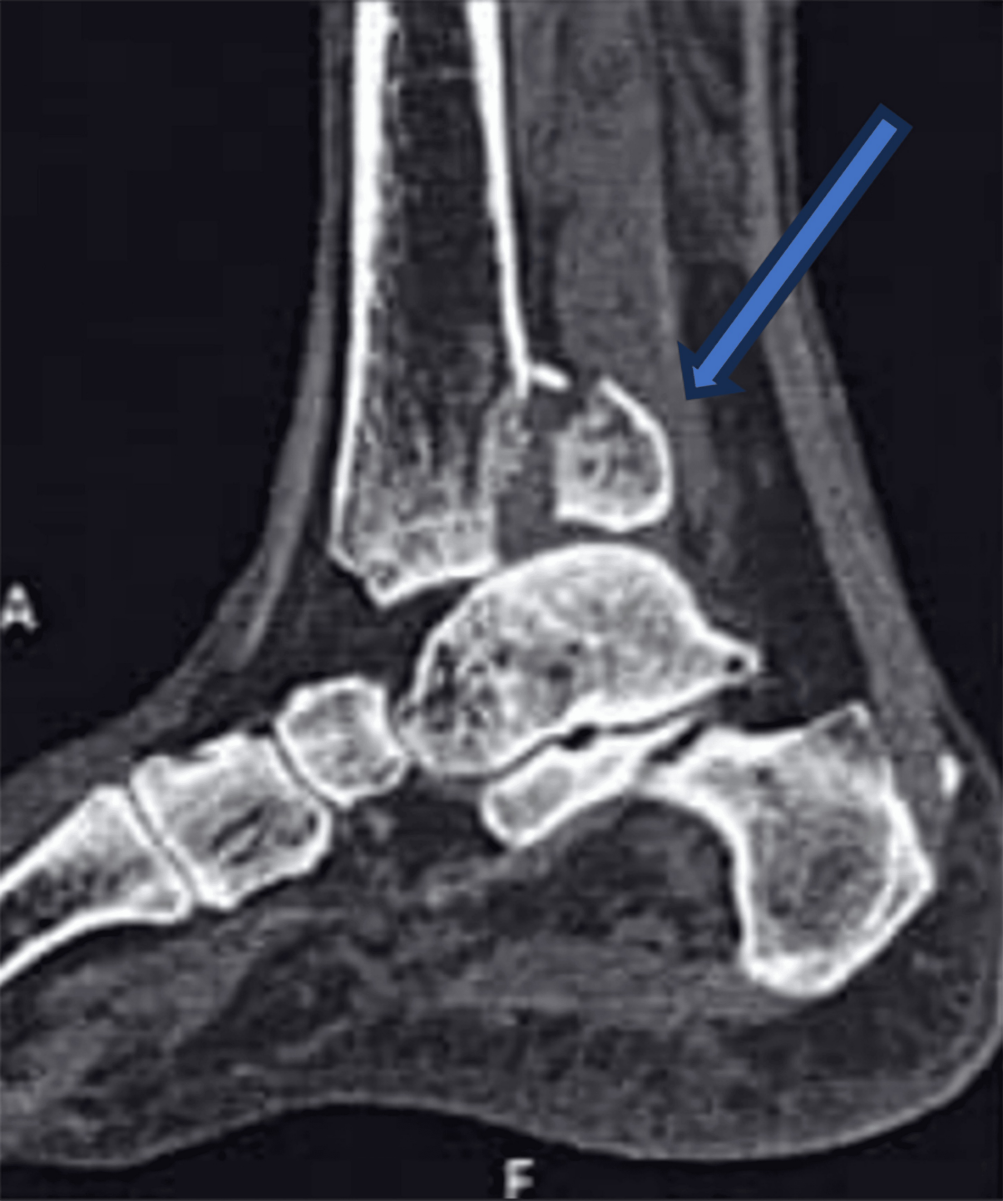
Sagittal section CT scan of an ankle A sagittal section of the CT scan of the ankle showing posterior malleolus fracture. CT: Computed tomography

**Figure 3:**
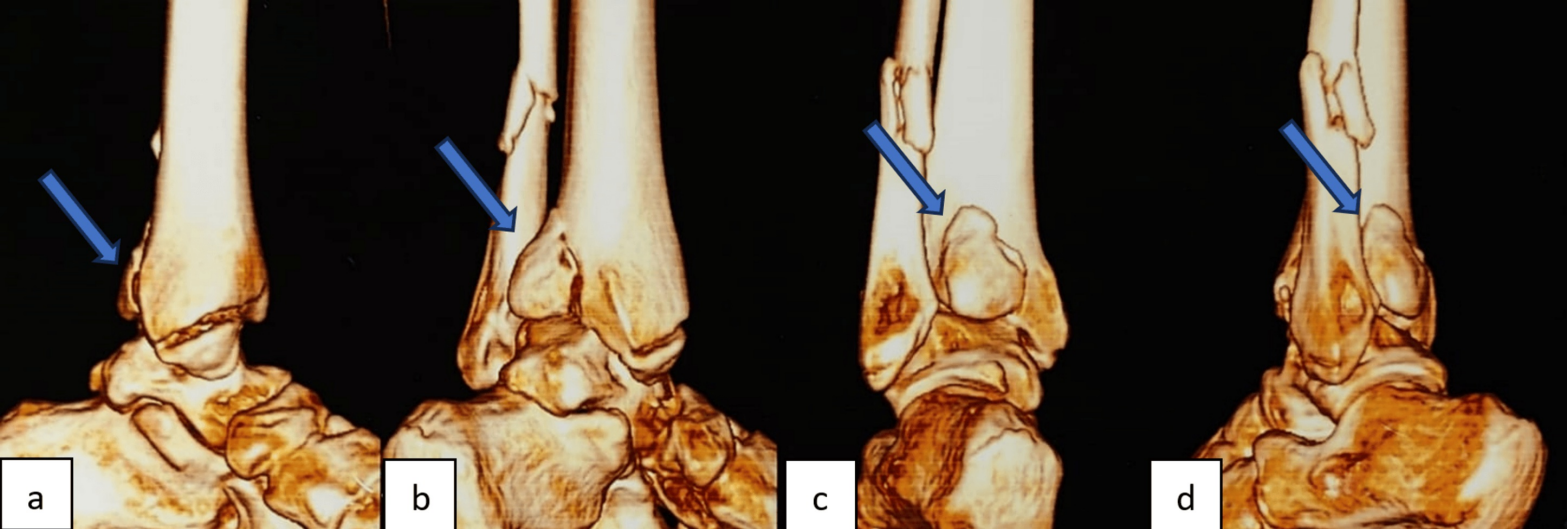
3D CT of an ankle showing posterior malleolus fracture fragment (a), (b), (c), and (d) showing different viewing angles of posterior malleolus fracture in 3D CT. CT: Computed tomography

**Figure 4:**
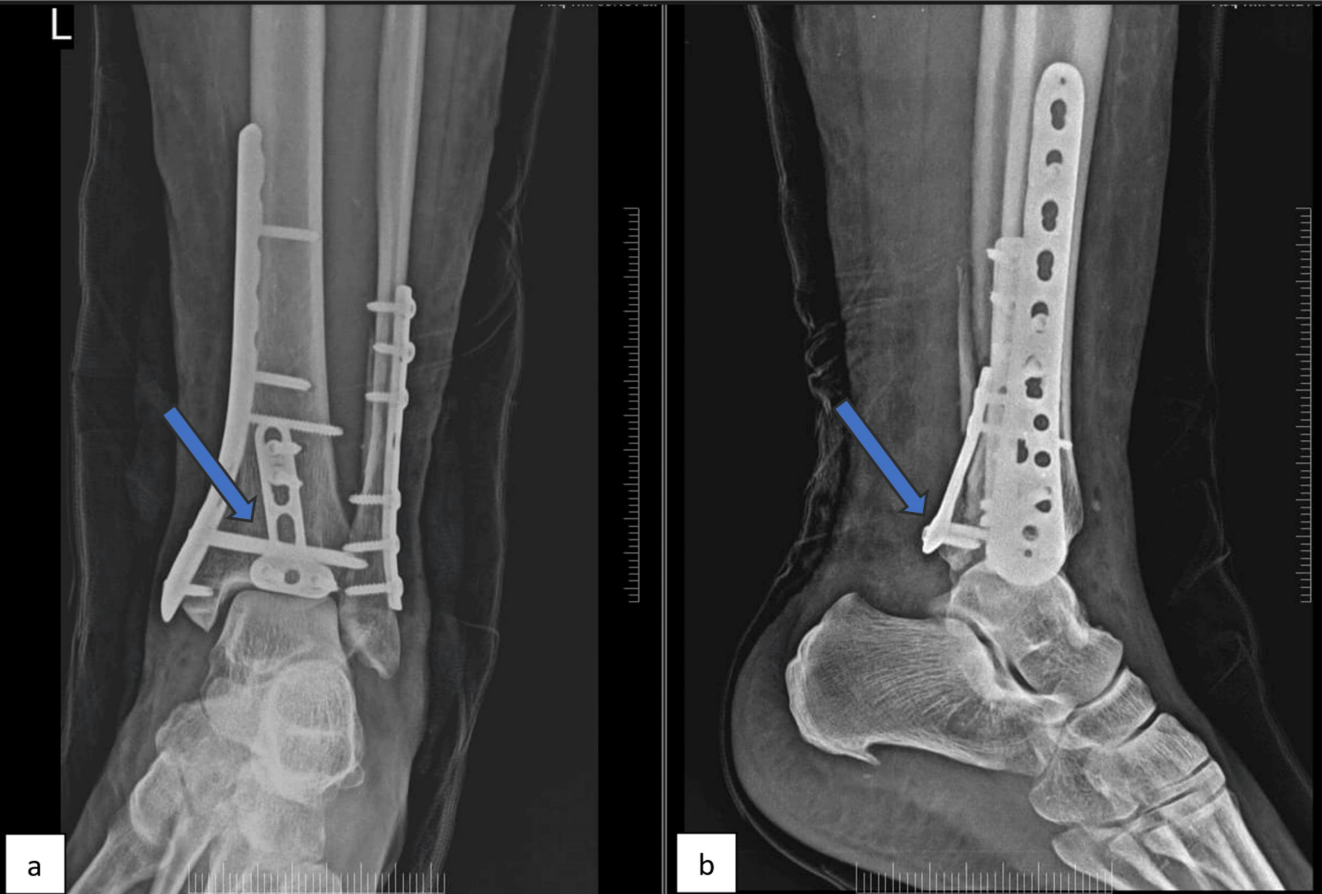
X-ray of an ankle in (a) anteroposterior and (b) lateral view This is a representative X-ray from the PM group showing the posterior malleolus fixed using a locking T-plate. PM: Posteromedial

**Figure 5:**
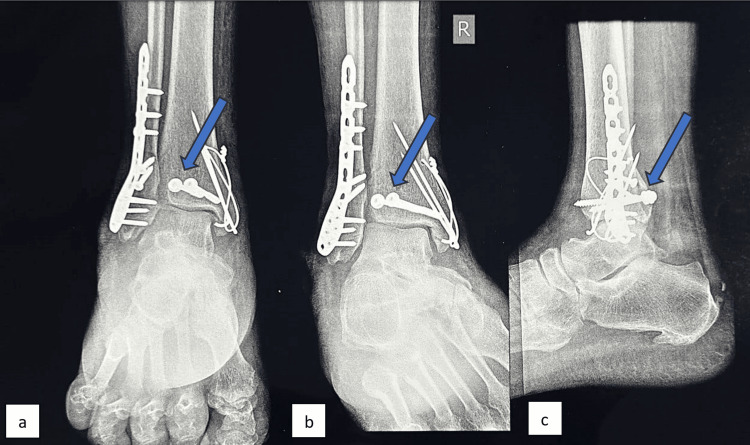
X-ray of an ankle in (a) anteroposterior, (b) mortise, and (c) lateral view This is a representative X-ray from the PL group where the posterior malleolus was fixed using two posteroanterior CC screws. PL: Posterolateral; CC: Cannulated cancellous

We evaluated the patients preoperatively with ankle X-rays in anteroposterior, lateral, and mortise views and CT scans (Figures 2-3). Fracture fragments of the posterior malleolus were reduced and fixed using buttress plates (Figure 4) or lag screws (Figure 5) based on the fragment size and surgeon's preference. After the surgery, patients were given a below-knee cast in a neutral position for three weeks, after which ROM exercises were initiated following cast removal. They were instructed to avoid bearing weight on the affected leg for six weeks. Patients were advised to start partial weight-bearing of 50% at six weeks, progressing to full weight-bearing by twelve weeks.

To assess outcomes, we used the AOFAS scores at six months, twelve months, and during the final follow-up. The AOFAS scores are widely used for assessing foot and ankle conditions. AOFAS scores combines patient-reported and clinician-determined items to evaluate pain, function, and alignment on a scale from 0 to 100. The score is divided into three main sections: pain (40 points), function (50 points), and alignment (10 points) [16]. Each section includes specific criteria, such as activity limitations, walking distance, gait abnormality, and stability [16]. We also evaluated operative time, arthritis, ankle ROM, infection, neurovascular injury, nonunion, and deep vein thrombosis. We compared the ROM of the affected ankle with that of the uninjured ankle. Postoperative X-rays were conducted to check for residual step-off or gaps in the articular reduction and arthritis using Bargon’s criteria at the final follow-up.

Continuous variable AOFAS scores were compared using independent t-tests at follow-ups. A P-value of less than 0.05 was considered statistically significant.

## Results

Patient demographics

The demographic analysis of the PL and PM groups highlighted several key differences. In the PL group, the age distribution was: four patients (19.04%) aged 18-30, three patients (14.29%) aged 31-40, three patients (14.29%) aged 41-50, five patients (23.81%) aged 51-60, and six patients (28.57%) aged 61-70. The gender distribution was nearly balanced, with 11 males (52.38%) and 10 females (47.62%). The leading cause of injury was falling from a height, accounting for eight patients (38.01%), followed by falls (six patients, 28.57%), motor vehicle accidents (five patients, 23.80%), and sports injuries (two patients, 9.52%).

In the PM group, the age distribution was: six patients (28.57%) aged 18-30, one patient (4.76%) aged 31-40, seven patients (33.33%) aged 41-50, six patients (28.57%) aged 51-60, and one patient (4.76%) aged 61-70. The gender distribution was also nearly equal, with 10 males (47.62%) and 11 females (52.38%). The predominant cause of injury was falling from a height, representing 14 patients (66.67%), followed by motor vehicle accidents (four patients, 19.04%), sports injuries (two patients, 9.52%), and falls (one patient, 4.76%).

Overall, both groups identified falls and motor vehicle accidents as significant causes of injury, but the PM group showed a markedly higher percentage of injuries from falls from height. The age and gender distributions reflect differing demographic profiles between the two groups.

Operative time (mins) distribution

The operative time distribution for PL and PM groups (Table 1) revealed notable differences. For the PL group, the majority of patients, 10 (47.62%), had surgeries lasting between 91 and 140 minutes. This was followed by seven patients (33.34%) with procedures lasting 50-90 minutes and four (19.04%) with surgeries ranging from 141-180 minutes. This distribution indicates that most PL patients experienced moderate-length operations, with fewer undergoing shorter or longer procedures. The mean ± standard deviation (SD) of the PL group was 107.38 ± 33.35.

**Table 1:** Operative time (mins) distribution in PL and PM groups The table represents the time taken to perform the posterolateral and posteromedial approaches. PL: Posterolateral; PM: Posteromedial; mins: Minutes

Operative Time (mins)	PL Patient Count	PL Percentage (%)	PM Patient Count	PM Percentage (%)
50-90	7	33.34	12	57.15
91-140	10	47.62	4	19.04
141-180	4	19.04	5	23.81
Total	21	100.00	21	100.00

In contrast, the PM group showed a different pattern; 12 patients (57.15%) had shorter operative times of 50-90 minutes, notably higher than the PL group. Only four patients (19.04%) had procedures lasting 91-140 minutes, and five patients (23.81%) had longer surgeries of 141-180 minutes. This suggests that PM patients are more likely to undergo shorter surgical procedures than PL patients, who generally experience more moderate-length surgeries. The mean ± SD of the PM group was 106.24 ± 39.51. The P-value was 0.9198.

Overall, the data highlights that PM patients typically have shorter operative times, while PL patients have a more balanced distribution across moderate to longer surgery durations.

Fragment size fixed (%) in the PL and PM groups

The distribution of fragment size fixed in the PL and PM groups revealed distinct patterns.

**Figure 6:**
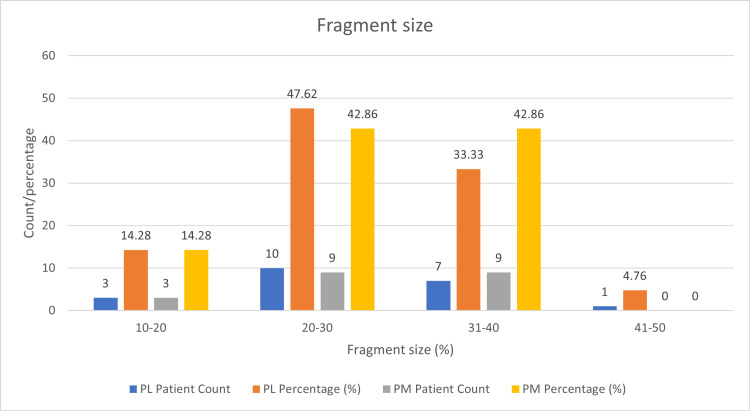
Fragment size fixed (%) in PL and PM groups The figure shows the percentage of tibial plafond fractures forming posterior malleolar fragments in both the PL and PM groups. PL: Posterolateral; PM: Posteromedial

In the PL group (Figure 6, Table 2), the majority of patients, 10 (47.62%), had a fragment size fixed between 20% and 30%. This was followed by seven patients (33.33%) with a fragment size fixed between 30% and 40%, three patients (14.28%) with a size fixed between 10% and 20%, and only one patient (4.76%) with a fragment size fixed between 40% and 50%. This indicates that half of the PL patients had moderate fragment fixation of 20-30%.

**Table 2:** Fragment size fixed (%) in PL and PM groups The table represents the percentage of tibial plafond fractures forming posterior malleolar fragments. PL: Posterolateral; PM: Posteromedial

Fragment size	PL Patient Count	PL Percentage (%)	PM Patient Count	PM Percentage (%)
10-20	3	14.28	3	14.28
20-30	10	47.62	9	42.86
31-40	7	33.33	9	42.86
41-50	1	4.76	0	0
Total	21	100.00	21	100.00

In the PM group (Figure 6, Table 2), the distribution was slightly different: nine patients (42.86%) had a fragment size fixed between both 20-30% and 31-40%, and three patients (14.28%) had a size fixed between 10% and 20%. Notably, no PM patients had a 41-50% fragment size.

Overall, while both groups predominantly have patients in the 20-30% and 31-40% fragment size categories, the PL group has a higher percentage in the 20-30% category and a lower percentage in the 31-40% category than the PM group. This reflects some variability in fragment size fixation practices between the two groups.

Loss of range of movements (degrees)

**Figure 7:**
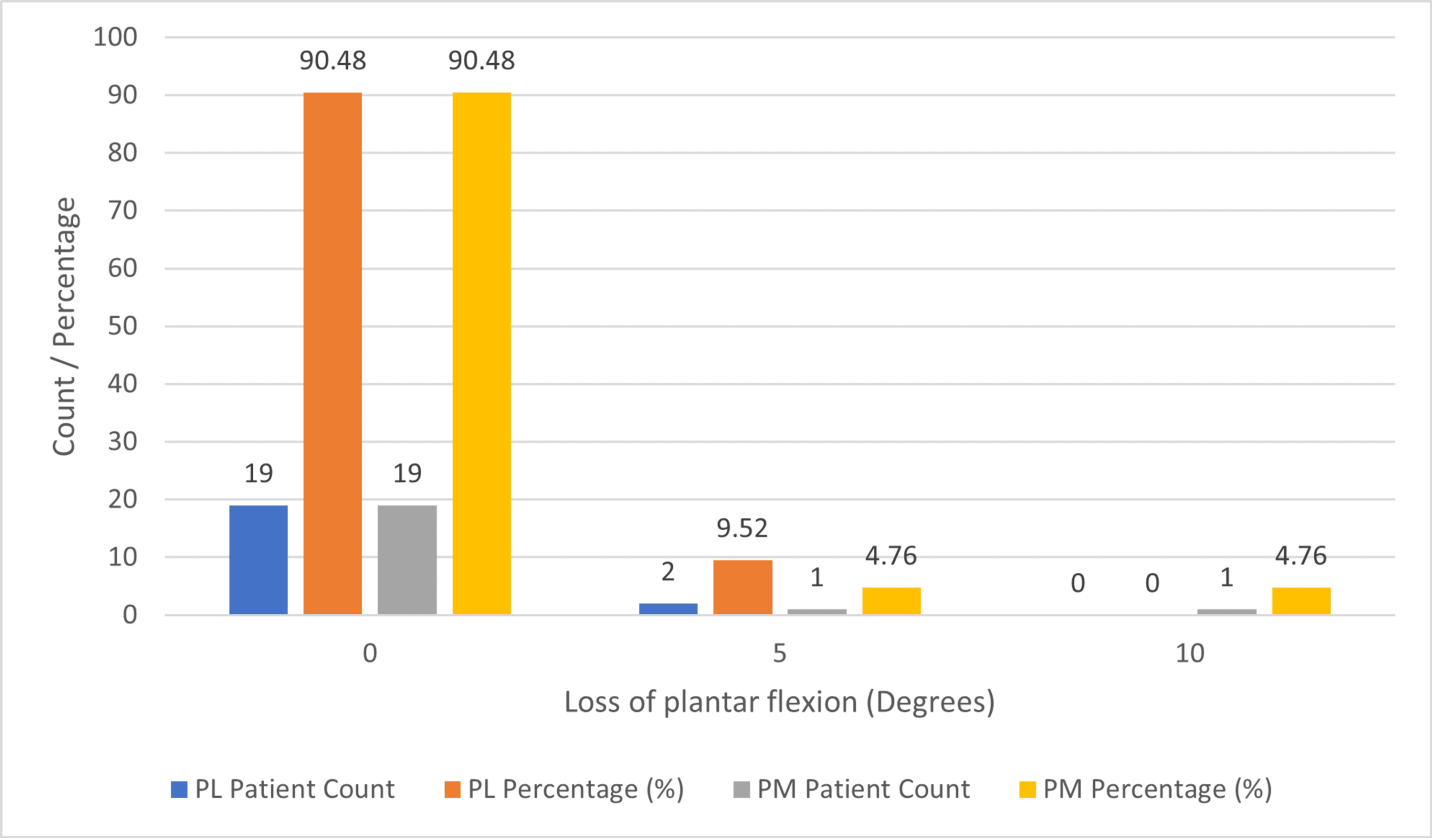
Loss of plantar flexion (degrees) in PL and PM groups The figure shows the loss of the affected ankle's plantar flexion (degrees) compared to the contralateral side at the end of the final follow-up. PL: Posterolateral; PM: Posteromedial

Most PL and PM patients exhibited no loss of plantar flexion (Figure 7), with 19 PL patients and 19 PM patients representing 90.48% of their respective groups.

**Figure 8:**
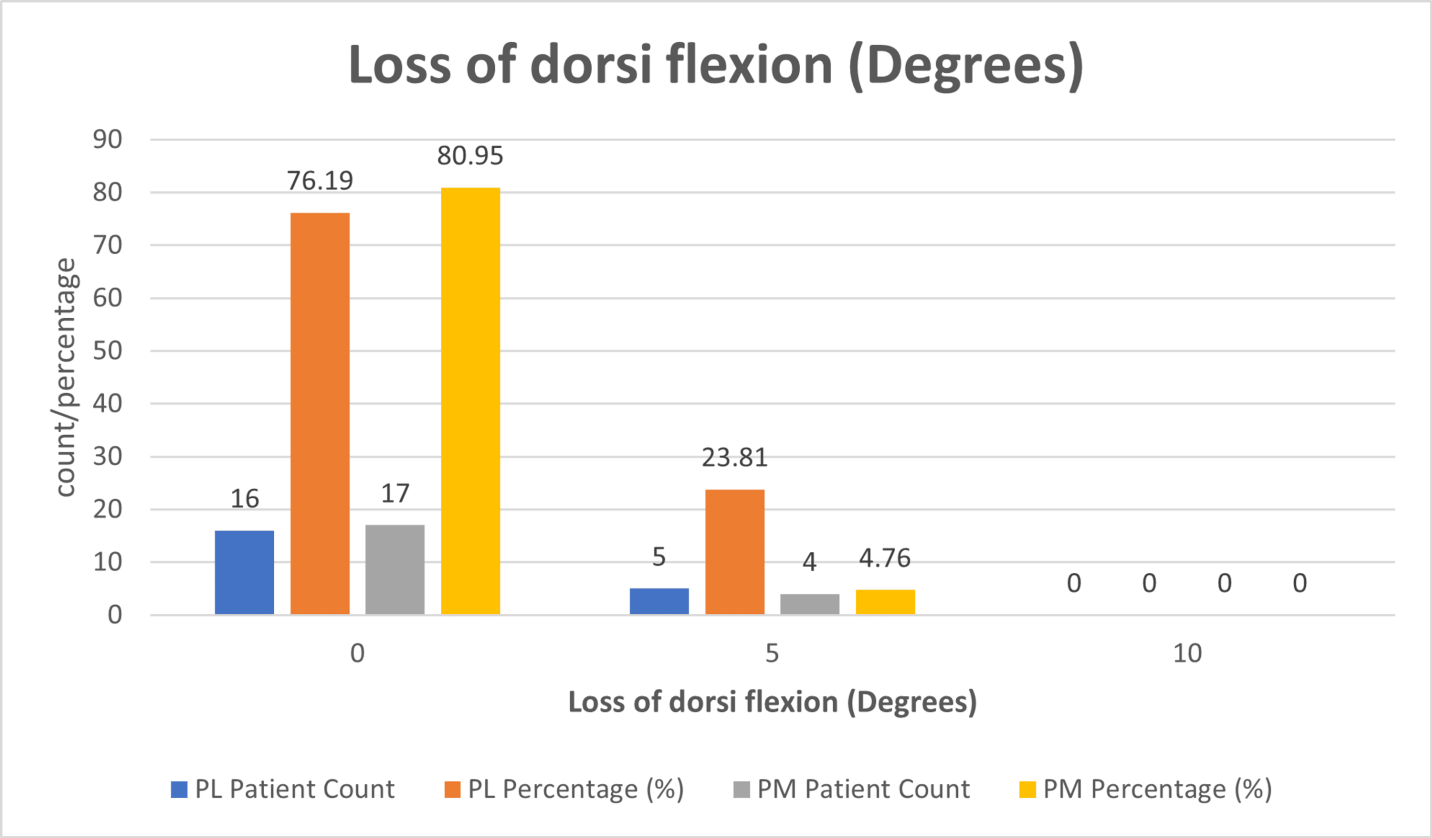
Loss of dorsi flexion (degrees) in PL and PM groups The figure shows loss of the affected ankle's plantar flexion (degrees) compared to the contralateral side at the end of the final follow-up. PL: Posterolateral; PM: Posteromedial

Among the remaining patients, two (9.52%) in the PL group and one (4.76%) in the PM group developed a five-degree loss of plantar flexion. Additionally, among PM patients, one (4.76%) suffered from a 10-degree loss of plantar flexion, whereas no PL patients fell into this category. Meanwhile, five (23.81%) patients in the PL group had a loss of dorsi flexion (Figure 8) of five degrees, and four (19.04%) patients had a loss of five degrees in the PM group.

Pilon fracture union and articular surface step-off

The fractures were reduced either by a lag screw or buttress plate based on the fracture fragment size and the surgeon's preference with a plan to keep the residual step-off less than 2 mm. During the follow-ups, ankle radiographs were taken to evaluate the fixation and step-off. Union was seen with a mean period of 3.9 months. There were no cases of delayed or nonunion, and three patients from the PL group and four from the PM group had step-off between 2 mm and 3 mm.

Arthritis as a complication

The results demonstrated that one case was observed in the PM group, which developed Bargon’s grade 2 arthritis as a complication with a step-off in the articular surface of the ankle joint between 2 mm and 3 mm. In contrast, there was no case of this complication in the PL group.

Infection

In our study, three cases, one from the PL group and two from the PM group, developed a superficial infection without necrosis or wound dehiscence.

AOFAS scores at six-month follow-up

At follow-up (Table 3), the clinical outcomes were assessed using the AOFAS scores. The results demonstrated that the PL group had a higher mean AOFAS score of 87.52 with an SD of 2.92. In contrast, the PM group had a mean score of 84.95 with an SD of 3.25. The P-value for the comparison was 0.01, indicating a statistically significant difference favoring the PL approach.

**Table 3:** AOFAS scores at six-month follow-up. The table represents the mean, SD, and P-value at six-month follow-up. P-value indicates that the difference between the two approaches is statistically significant, favoring the PL approach. AOFAS: American Orthopedic Foot and Ankle Society; SD: Standard deviation; PL: Posterolateral; PM: Posteromedial

	Mean	SD	P value
PL	87.52	2.92	0.01^*^
PM	84.95	3.25	

The findings suggest that the PL approach provided superior clinical outcomes compared to the PM approach at the six-month follow-up. The statistically significant difference in AOFAS scores highlights the potential benefits of the PL approach in achieving better postoperative results.

AOFAS scores at 12-month follow-up

The results (Table 4) demonstrated that the PL group had a higher mean AOFAS score of 90.28 with an SD of 1.72. In contrast, the PM group had a mean score of 88.86 with an SD of 2.41. The P-value for the comparison was 0.034, indicating that the difference between the two approaches was statistically significant, favoring the PL approach.

**Table 4:** AOFAS scores at 12-month follow-up The table represents the mean, SD, and P-value at 12-month follow-up. P-value indicates that the difference between the two approaches is statistically significant, favoring the PL approach. AOFAS: American Orthopedic Foot and Ankle Society; SD: Standard deviation; PL: Posterolateral; PM: Posteromedial

	Mean	SD	P value
PL	90.28	1.72	0.034^*^
PM	88.86	2.41	

The difference in AOFAS scores at the 12-month follow-up was statistically significant; the data suggest that the PL approach offers better clinical outcomes than the PM approach at the 12-month follow-up. The PL approach's higher mean score and lower SD indicate more consistent and potentially favorable results.

AOFAS scores at final follow-up

The mean ± SD (range in months) of the final follow-up period of the PL and PM groups were 20.57 ± 2.7 (14-24) and 20.48 ± 2.54 (16-24) months, respectively. The results (Table 5) demonstrate that the PL group had a higher mean AOFAS score of 92.48 with an SD of 1.53. In contrast, the PM group had a mean score of 91.43 with an SD of 2.38. The P-value for the comparison was 0.97, indicating that the difference between the two approaches was not statistically significant.

**Table 5:** AOFAS scores at final follow-up The table epresents the final follow-up's mean, SD, and P-value. P-value indicates that the difference between the two approaches is not statistically significant. AOFAS: American Orthopedic Foot and Ankle Society; SD: Standard deviation; PL: Posterolateral; PM: Posteromedial

	Mean	SD	P value
PL	92.48	1.53	0.097
PM	91.43	2.38	

Although the difference in AOFAS scores at the final follow-up was not statistically significant, the data suggests that the PL approach may offer slightly better clinical outcomes than the PM approach. The PL approach's higher mean score and lower SD indicate more consistent and potentially favorable results.

## Discussion

Age group distribution comparison

In our study, the mean age of patients in the PL group was 47.76 ± 17.07 years, ranging from 19-71 years. The PM group's mean age was 43.29 ± 14.67 years, ranging from 18-69 years. These findings are similar to those reported by Zhong *et al*. (2017), who found that the PL group had a mean age of 42.7 ± 12.3 years (range: 18-63 years) and the PM group had a mean age of 44.2 ± 13.4 years (range: 18-64 years) [1]. Our study indicates a slightly older demographic for the PL approach.

In the PL group, the distribution across age groups was as follows: four patients (19.04%) in the 18-30 age group, three patients (14.29%) in the 31-40 group, four patients (19.04%) in the 41-50 group, 23.81% in the 51-60 group, and six patients (28.57%) in the 61-70 group.

For the PM group, the youngest age group (18-30) had the highest proportion with six patients (28.57%). This was followed by a notable drop to one patient (4.76%) in the 31-40 group, the highest number in the 41-50 age group with seven patients (33.33%), six patients (28.57%) in the 51-60 group, and a low number with one patient (4.76%) in the 61-70 group.

Gender distribution comparison

In our study, the PL group consisted of 11 males (52.38%) and 10 females (47.62%), indicating a slight male predominance. Conversely, the PM group had 10 males (47.62%) and 11 females (52.38%), showing a slight female predominance. Zhong *et al*. (2017) reported a different trend; their study revealed a higher percentage of females in the PL group (20 out of 28) and an equal distribution of males and females in the PM group (10 each) [1].

Cause of injury distribution comparison

In our study, 'fall from height' was the most common cause of injury for the PL group, accounting for eight patients (38.01%). Other causes included 'fall at ground level' (six patients, 28.57%) and 'motor vehicle accidents' (five patients, 23.80%). Sports injuries were the least common with two patients (9.52%). In comparison, Zhong *et al.* (2017) reported 'fall at ground level' as the most common cause (57.1%) and 'motor vehicle accidents' as a notable cause (25%) [1].

For the PM group, 'fall from height' was also the leading cause in our study with 14 patients (66.67%). Other causes included 'motor vehicle accidents' (four patients, 19.04%) and sports injuries (two patients, 9.52%). Zhong *et al*. (2017) reported 'fall at ground level' as the most common cause for PM patients (75%) and 'motor vehicle accidents' as 15% [1].

This comparative analysis highlights the significant role of 'fall from height' and 'fall at ground level' in causing posterior malleolus fractures treated with either surgical approach.

Operative time (mins) distribution comparison

The operative time distribution for patients who underwent PL and PM approaches for posterior malleolus fixation in trimalleolar fractures between the present study and the study by Ceccarini *et al*. (2024) [6]. In our study, in the PL patient group, the majority, 10 (47.62%), underwent operations of 91-140 minutes, but in the PM group, a significant portion, 12 (57.15%), had shorter operative times of 50-90 minutes. The mean operative time in the PL group was 107.38 ± 33.35. minutes; in the PM group, it was 106.24 ± 39.51. The P-value was 0.9198.

The PL approach tends to have more operative time, while the PM approach often results in shorter operative times [6]. This can be attributed to extensive exposure to reach the fracture fragment and the risk of injury to the sural nerve in the PL approach [6]. However, in our study, the mean operating time in both groups was almost the same without any statistically significant difference. Literature comparing the operative time in the PL and PM approaches is unavailable, and the operative time can vary depending on the surgeon’s familiarity with the approach.

Loss of range of movements (degrees)

Two patients in the PL group (9.52%) and one PM patient (4.76%) developed a five-degree loss of plantar flexion. Additionally, among PM patients, one (4.76%) suffered from a 10-degree loss of plantar flexion. Meanwhile, five (23.81%) patients in the PL group had a loss of dorsi flexion of five degrees, and four (19.04%) patients had a loss of five degrees in the PM group. In contrast, in a study by Ceccarini *et al.*, 7.4% of patients developed a loss of range of movements of 15 degrees [6]. In the study by Zhong *et al.* (2017), the median loss of dorsi flexion was 0 degrees, with the 75th percentile (P75) equal to five and the 25th percentile (P25) equal to 0, in both PM and PL groups. However, the median loss of plantar flexion was 0, with P25 equal to 0 and P75 equal to 0, indicating only a few patients developed loss of dorsi flexion in both PL and PM, with no statistical difference between them.

Comparison of fragment size fixed

In the present study, the mean fragment size operated in the PL patient group was 27.67 ± 7.47% and the mean fragment size operated in the PM patient group was 25.04 ± 4%. This suggests that the fragment size fixed in the PM approach had a lower fragment size fixed compared to the PL group. This was comparable to a study by Zhong *et al*., where the mean percentage of fragments fixed in the PM group was 25.4 ± 12.5%, but the mean percentage of fragments fixed in the PL group was much lower, 20.9 ± 7.9%, than in our study [1].

Pilon fracture union and articular surface step-off

In our study, there were no cases of delayed or nonunion, and three patients from the PL group and four from the PM group had step-off between 2 mm and 3 mm. In a study by Ceccarini *et al*., among the patients who were operated on with a buttress plate or lag screws with a PL approach, they observed that 21 patients had a step-off of more than 2 mm [6]. Among 48 patients in a study by Zhong *et al*., there were no cases of nonunion, and there was one case in each PM and PL group with a step-off between 2 mm and 3 mm [1]. Zhou *et al*. reported that the bony union was seen at the mean period of 3.8 months in their PL group [17].

Arthritis

In a study by Ruokun *et al.*, among 32 patients, one patient in the PM group developed arthritis [18]. In another study by Zhong *et al*., among 20 cases by PM and 28 by PL approach, two in the PL group and one in the PM group developed arthritis [1]. However, our study had only one case of arthritis in the PM group with an articular surface step-off of 2-3 mm. The majority of surgeons believe that to prevent posttraumatic OA of the ankle joint and to create a stable ankle, fragments of posterior malleolus, including more than 25-30% of the distal tibial plafond surface, should be corrected [5]. Poor reduction of ankle fractures can lead to early onset OA, resulting in functional impairment and ankle joint pain [11]. Joint step-off has been recognized as a significant predictor of OA onset, yet its influence on functional outcomes remains a topic of ongoing discussion [6].

Infection

In our study, three cases, one from the PL group and two from the PM group, developed a superficial infection without necrosis or wound dehiscence. The infection was resolved with active treatment using antibiotics and dressings. In a study by Zhong *et al*., there was one case each of superficial infection in the PM and PL groups and one case of wound dehiscence in the PM group [1]. Ruokun *et al*. reported that there was only one superficial infection among 38 patients in the PL group [18].

AOFAS scores at six-month follow-up

In the PL patient group, the present study's mean AOFAS score was 87.52 ± 2.92 and the AOFAS score of the PM patient group was 84.95 ± 3.25, with a P-value of 0.01, which is statistically significant, favoring the PL approach. In contrast, in the study by Zhong *et al*., at six-month follow-up, the PL group AOFAS score was 89.86 ± 6.40 and that of the PM group was 91.35 ± 5.69, which was not statistically significant [1].

In a study by Lei Yang *et al*., the average AOFAS score was 81.35 ± 6.15 six months after surgery. It was mentioned that the PL technique has been suggested in treating ankle fractures involving the posterior malleolus and several trials have shown promising outcomes [2].

AOFAS scores at 12-month follow-up

In the PL patient group, the present study's mean AOFAS score was 90.28 ± 1.72 and the AOFAS score of the PM patient group was 88.86 ± 2.41, with a P-value of 0.034, which is statistically significant, favoring the PL approach. In contrast, in the study by Zhong *et al*., at 12-month follow-up, the PL group AOFAS score was 91.36 ± 5.88 and that of the PM group was 92.45 ± 4.43, which was not statistically significant [1].

Overall, the comparison demonstrates that both the present study and Wang *et al*.'s findings are consistent in showing that PL patients tend to have a higher percentage of excellent outcomes, while PM patients predominantly achieve good outcomes. This reinforces the effectiveness of both surgical approaches in managing posterior malleolus fractures, with slight variations in the distribution of the highest functional scores [1].

AOFAS scores at final follow-up

In the PL patient group, the present study's mean AOFAS score was 92.48 ± 1.53 and the AOFAS score of the PM patient group was 91.43 ± 2.38, with a P-value of 0.097, which is statistically not significant, indicating that in the final follow-up there was no significant difference between the PL and PM groups. This is in line with the study by Zhong *et al*.; at the final follow-up, the PL group AOFAS score was 91.93 ± 5.87 and that of the PM group was 92.85 ± 4.42, which was not statistically significant [1].

In the study by Lei Yang *et al*., the average AOFAS score in the PL group was 90.56 ± 4.98 at the final follow-up, similar to the mean in our study [2].

The comparison demonstrates that the present study and Zhong *et al*.'s findings consistently showed that PL patients tend to have a higher percentage of excellent outcomes, while PM patients predominantly achieve good outcomes with a notable proportion reaching excellent scores [1]. This reinforces the effectiveness of both surgical approaches in managing posterior malleolus fractures with slight variations in the distribution of the highest functional scores.

The superior outcomes observed with the PL approach may be attributed to several factors. The PL approach offers better anatomical access to the posterior malleolus, allowing for more precise reduction and fixation. This could result in a more stable construct, explaining the improved AOFAS scores and range of motion in the early postoperative period. The PL approach may involve less soft tissue disruption and provide better visualization, leading to a more effective surgical technique and potentially quicker rehabilitation [1,19].

Limitations

Despite the promising findings, this study has several limitations. The relatively small sample size may limit the generalizability of the results to a broader population. Additionally, postoperative CT scans were not done. The generalizability of the findings is further limited by the specific characteristics of the study population, which may not fully represent the diversity of patients with trimalleolar fractures. Finally, the outcomes could be influenced by the surgeons' familiarity and experience with the PL and PM approaches, potentially introducing bias in favor of one technique over the other. Future studies should explore the potential for combining both approaches in complex cases and investigate the role of surgeon experience in the success of each technique.

## Conclusions

In conclusion, the study highlights the potential benefits of the PL approach for the fixation of posterior malleolus in trimalleolar fractures, particularly regarding early postoperative outcomes and complication rates. The improved visualization and precision offered by the PL approach may contribute to better initial functional recovery and a lower incidence of complications such as OA. However, long-term follow-up data suggest that the PL and PM approaches are equally effective in achieving satisfactory clinical outcomes, emphasizing the importance of individualized treatment planning based on specific fracture characteristics and patient factors. Future studies with a larger sample size and a more extended follow-up period are necessary to validate these findings further and optimize the surgical management of posterior malleolus fractures. Further prospective studies and randomized controlled trials are needed to reinforce this conclusion.

## References

[1] Zhong S, Shen L, Zhao JG (2017). Comparison of posteromedial versus posterolateral approach for posterior malleolus fixation in trimalleolar ankle fractures. Orthop Surg.

[2] Yang L, Yin G, Zhu J (2023). Posterolateral approach for posterior malleolus fixation in ankle fractures: functional and radiological outcome based on Bartonicek classification. Arch Orthop Trauma Surg.

[3] Kumar SG, Lu J, Ratish S, Arjun S, Sundar K, Liang J (2019). Treatment of posterior malleolus fracture through posterolateral approach. Open J Orthop.

[4] Köken M, Akşahin E, Çelebi L (2016). Current concepts in posterior malleol fractures. (Article in Turkish). Acta Orthop Traumatol Turc.

[5] Abdelgawad AA, Kadous A, Kanlic E (2011). Posterolateral approach for treatment of posterior malleolus fracture of the ankle. J Foot Ankle Surg.

[6] Ceccarini P, Donantoni M, Milazzo F (2024). Fixation of posterior malleolus in trimalleolar ankle fractures: anteroposterior screw or posterolateral plate?. Appl Sci.

[7] Langenhuijsen JF, Heetveld MJ, Ultee JM, Steller EP, Butzelaar RM (2002). Results of ankle fractures with involvement of the posterior tibial margin. J Trauma.

[8] Haraguchi N, Haruyama H, Toga H, Kato F (2006). Pathoanatomy of posterior malleolar fractures of the ankle. J Bone Joint Surg Am.

[9] Hartford JM, Gorczyca JT, McNamara JL, Mayor MB (1995). Tibiotalar contact area. Contribution of posterior malleolus and deltoid ligament. Clin Orthop Relat Res.

[10] Court-Brown CM, McBirnie J, Wilson G (1998). Adult ankle fractures - an increasing problem?. Acta Orthop Scand.

[11] Huber M, Stutz PM, Gerber C (1996). Open reduction and internal fixation of the posterior malleolus with a posterior antiglide plate using a postero-lateral approach - a preliminary report. Foot Ankle Surg.

[12] Court-Brown CM (2003). Skeletal trauma: basic science, management and reconstruction. J Bone Joint Surg Br.

[13] Erdem MN, Erken HY, Burc H, Saka G, Korkmaz MF, Aydogan M (2014). Comparison of lag screw versus buttress plate fixation of posterior malleolar fractures. Foot Ankle Int.

[14] Vacas-Sánchez E, Olaya-González C, Abarquero-Diezhandino A, Sánchez-Morata E, Vilá-Rico J (2020). How to address the posterior malleolus in ankle fractures? A decision-making model based on the computerised tomography findings. Int Orthop.

[15] Bois AJ, Dust W (2008). Posterior fracture dislocation of the ankle: technique and clinical experience using a posteromedial surgical approach. J Orthop Trauma.

[16] Van Lieshout EM, De Boer AS, Meuffels DE, Den Hoed PT, Van der Vlies CH, Tuinebreijer WE, Verhofstad MH (2017). American Orthopaedic Foot and Ankle Society (AOFAS) ankle-hindfoot score: a study protocol for the translation and validation of the Dutch language version. BMJ Open.

[17] Zhou Q, Lu H, Wang Z, Yu S, Zhang H (2017). Posterolateral approach with buttress plates and cannulated screw fixation for large posterior malleolus fractures. J Foot Ankle Surg.

[18] Ruokun H, Ming X, Zhihong X, Zhenhua F, Jingjing Z, Kai X, Jing L (2014). Postoperative radiographic and clinical assessment of the treatment of posterior tibial plafond fractures using a posterior lateral incisional approach. J Foot Ankle Surg.

[19] Wang C, Huang S, Wang Z, Sun X, Lin C, Li Q, Wang Y (2017). Clinical characteristics and surgical strategy of pilon fracture with different injury mechanisms. (Article in Chinese). Chin J Anat Clin.

